# Surface Albedo and Temperature Models for Surface Energy Balance Fluxes and Evapotranspiration Using SEBAL and Landsat 8 over Cerrado-Pantanal, Brazil

**DOI:** 10.3390/s21217196

**Published:** 2021-10-29

**Authors:** Lucas Peres Angelini, Marcelo Sacardi Biudes, Nadja Gomes Machado, Hatim M. E. Geli, George Louis Vourlitis, Anderson Ruhoff, José de Souza Nogueira

**Affiliations:** 1 Instituto Federal Goiano, km 01, Rodovia Sul Goiana, Rio Verde 75901-970, Brazil; lucas.angelini@ifgoiano.edu.br; 2 Physics Institute, Universidade Federal de Mato Grosso, 2367 Av. Fernando Corrêa da Costa, Cuiabá 78060-900, Brazil; nogueira@ufmt.br; 3 Instituto Federal de Mato Grosso, Av. Juliano da Costa Marques, Cuiabá 78050-560, Brazil; nadja.machado@blv.ifmt.edu.br; 4 New Mexico Water Resources Institute and Department of Animal and Range Sciences, New Mexico State University, Las Cruces, NM 88003, USA; 5 Biological Sciences Department, California State University San Marcos, 333 S. Twin Oaks Valley Rd., San Marcos, CA 92096, USA; georgev@csusm.edu; 6 Institute of Hydraulic Research, Universidade Federal do Rio Grande do Sul, 9500 Av. Bento Gonçalves, Porto Alegre 91501-970, Brazil; anderson.ruhoff@ufrgs.br

**Keywords:** performance, land surface temperature, atmospheric correction, flux towers

## Abstract

The determination of the surface energy balance fluxes (SEBFs) and evapotranspiration (ET) is fundamental in environmental studies involving the effects of land use change on the water requirement of crops. SEBFs and ET have been estimated by remote sensing techniques, but with the operation of new sensors, some variables need to be parameterized to improve their accuracy. Thus, the objective of this study is to evaluate the performance of algorithms used to calculate surface albedo and surface temperature on the estimation of SEBFs and ET in the Cerrado-Pantanal transition region of Mato Grosso, Brazil. Surface reflectance images of the Operational Land Imager (OLI) and brightness temperature ($T_{b}$) of the Thermal Infrared Sensor (TIRS) of the Landsat 8, and surface reflectance images of the MODIS MOD09A1 product from 2013 to 2016 were combined to estimate SEBF and ET by the surface energy balance algorithm for land (SEBAL), which were validated with measurements from two flux towers. The surface temperature ($T_{s}$) was recovered by different models from the $T_{b}$ and by parameters calculated in the atmospheric correction parameter calculator (ATMCORR). A model of surface albedo ($a_{sup}$) with surface reflectance OLI Landsat 8 developed in this study performed better than the conventional model ($a_{con}$) SEBFs and ET in the Cerrado-Pantanal transition region estimated with $a_{sup}$ combined with $T_{s}$ and $T_{b}$ performed better than estimates with $a_{con}$. Among all the evaluated combinations, SEBAL performed better when combining $a_{sup}$ with the model developed in this study and the surface temperature recovered by the Barsi model ($T_{s_{barsi}}$). This demonstrates the importance of an $a_{sup}$ model based on surface reflectance and atmospheric surface temperature correction in estimating SEBFs and *ET* by SEBAL.

## 1. Introduction

Surface energy balance fluxes (SEBFs) are one of the most important biophysical processes in environmental and hydrological studies [1,2,3]. SEBFs represent the processes of partitioning of available energy on the surface, measured by the net radiation (Rn), to evapotranspiration (*ET*) and soil and air heating, represented by soil heat flux (G) and sensible heat flux (H), respectively [1]. Among these SEBFs components, *ET* is widely studied due to its importance in climatic, hydrological, and agronomic strategy models [4].

In recent years, SEBFs and ET have been estimated from orbital satellite data, which require little meteorological data and generate reliable estimates at local and regional scales [4,5]. Among the most used models, the surface energy balance algorithm for land (SEBAL) has been successfully applied in different climatic regions and land covers [6]. SEBAL integrates orbital and meteorological data to compute SEBFs and *ET* [7].

Surface temperature ($T_{s}$) and surface albedo ($a_{sup}$) play an important role in estimating SEBFs and *ET* by SEBAL [8,9]. Rn is estimated by the radiation balance equation using surface meteorological data and obtained by remote sensors, such as surface reflectance and thermal radiance that makes it possible to estimate $a_{sup}$ and recover $T_{s}$, respectively [10]. H is calculated from an empirical linear relationship between the temperature gradient (dT) and $T_{s}$, considering two extreme conditions of water availability on the surface [8,11], while G is estimated by an empirical equation based on Rn, the normalized difference vegetation index (NDVI), $a_{sup}$, and $T_{s}$ [12,13]. Finally, the latent heat flux (LE) is estimated as a residue of the energy balance equation [8].

In the current formulation of SEBAL, SEBFs and *ET* are estimated by the conventional surface albedo ($a_{con}$) equation estimated by the planetary albedo ($a_{TOA}$) and corrected by atmospheric albedo, transmittance, and the brightness temperature ($T_{b}$), without atmospheric and surface emissivity correction [8,9,10,11]. Some variations of SEBAL, such as mapping evapotranspiration with internalized calibration (METRIC), include the atmospheric correction of the surface reflectance of the thermal band [11,14,15,16]. However, few studies have evaluated the combined effects of $a_{sup}$ and $T_{s}$ recovery on SEBAL and *ET* estimates by SEBAL. $a_{sup}$ is a key parameter in SEBF models, and its estimation under different atmospheric and surface conditions represents a major challenge [17,18]. Generally, the accuracy of $a_{sup}$ models varies between 10% and 28%, which suggests the need for their parameterization [18]. The $a_{sup}$ models based on surface reflectance were parameterized for TM, ETM, and MODIS sensors [19,20], but not for the OLI Landsat 8 sensor. This limits the estimation of $a_{sup}$ at a high spatial resolution after the discontinuation of the Landsat 5 satellite in 2011. The $a_{sup}$ models developed by [21] have been used in several studies on the dynamics of mass and energy of water bodies [22], the effect of biomass burning on meteorological parameters [23,24], urban climate and thermal comfort [25], and SEBFs and *ET* by SEBAL [6,26].

The recovery of $T_{s}$ by thermal radiance, corrected for the effects of the atmosphere and the surface emissivity, has been performed with errors smaller than 1 K [16,27]. Several algorithms have been developed to correct the attenuating effects of the atmosphere in the thermal band of TM (Thematic Mapper), ETM (Enhanced Thematic Mapper), and TIRS (Thermal Infrared Sensor) sensors [28,29,30]. These algorithms are based on the radioactive transfer equation, which relates the upward and downward flows of thermal radiance, atmosphere transmissivity, and surface emissivity to the thermal band [31,32,33].

A series of models for recovery of $T_{s}$ were developed for the TM, ETM, and TIRS sensors, with emphasis on the single-channel (SC) and split-window (SW) algorithms [34], the radiative transfer equation (RTE) [35], and the model developed by [29]. The SC model stands out for allowing the thermal band correction using atmospheric functions obtained from moderate resolution atmospheric transmission (MODTRAN) or through approximations resulting from a second order polynomial relationship with the atmospheric water vapor content [34,35]. The SW model starts from the premise that the attenuation of thermal radiance by atmospheric radiation is proportional to the difference in thermal radiance measured simultaneously at two different wavelengths [34]. Both the RTE models and the one developed by Barsi remove the effect of the atmosphere from thermal radiance by the radiative transfer equation, but the RTE model recovers $T_{s}$ through Plank’s inverse equation [35] and Barsi’s model by the equation calibrated for the sensor TIRS [29,36].

These models require some parameters obtained with the aid of radiosondes, which makes their wide application difficult [32]. An alternative to obtain atmospheric input parameters for $T_{s}$ recovery models was developed by NASA, using the atmospheric correction parameter calculator (ATMCORR). ATMCORR stands out for its simple operation, considering that its platform is online and requires only some meteorological data and for its robustness, and it can be applied for TM, ETM sensors, and TIRS in different latitudes over long periods [29,36]. This online platform integrates radioactive transfer codes (MODTRAN v4.0) with data from the National Centers for Environmental Prediction (NCEP) [29,36].

Given the importance of estimating SEBFs and *ET* from the $a_{sup}$, which is in turn estimated by the surface reflectance and the $T_{s}$ without atmosphere and the emissivity corrections, the objective of this study is to evaluate the performance of the $a_{sup}$ and $T_{s}$ recovery models for the estimation of SEBFs and *ET* by SEBAL in the Cerrado-Pantanal transition region of the state of Mato Grosso, Brazil. This transition zone consists of upland cerrado vegetation that grades into an extensive wetland complex, with natural woodlands, forests, grasslands, and human land covers, such as agriculture, pasture, and urban areas, which affect Ts, albedo, and other local climatic variables that are important for SEBFs and *ET* [1].

## 2. Materials and Methods

### 2.1. Study Site

**Figure 1:**
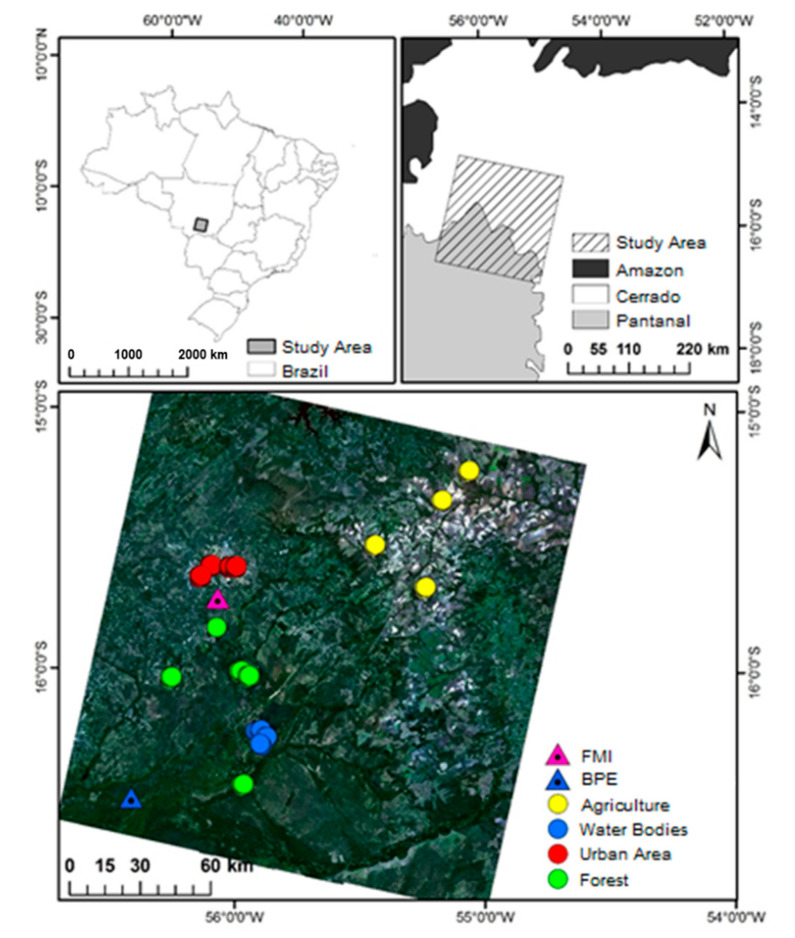
Location of the study area (**top-left**), location of the Cerrado-Pantanal transition region of Mato Grosso, Brazil (**top-right**), and the sample points (circles) and the flux towers in Fazenda Miranda (FMI) and Baía das Pedras (BPE) (**bottom**).

The study area is in the transition region Cerrado-Pantanal, covering path 226 and row 71 of the satellite Landsat 8 in southern Mato Grosso, Brazil (Figure 1). Data from two flux towers were used, one in the Cerrado and the other in the Pantanal. The Cerrado tower is located in Fazenda Miranda (FMI) (15°43′55″ S; 56°4′19″ W), approximately 15 km south of the city of Cuiabá. The vegetation at the FMI is dominated by native and exotic grasses and by the semi-deciduous trees *Curatella americana* L. and *Diospyros hispida* A.DC [37], and the soil is classified as Plinthosols [38]. The Pantanal flux tower is in the Baia das Pedras (BPE) of the Estância Ecológica SESC-Pantanal (16°29′52″ S; 56°24′44″ W), in the municipality of Poconé, approximately 160 km from Cuiabá. The predominant vegetation in BPE is composed of the tree *Combretum lanceolatum* Pohl [39], and the soil is classified as Gleysols [38]. The BPE topography is flat, with flooding occurring from January to June. The Köppen–Geiger climate classification of the entire study region is Aw [40]. Annual rainfall is 1372 mm, with a dry season from May to September and a wet season from October to April, and average annual temperature of 26.9 °C [41].

Four types of land uses (agriculture, urban areas, forest, and water bodies) were sampled in the study area to develop a surface albedo model using surface reflectance from the OLI Landsat 8 (Figure 1). The types of coverage were strategically selected because they represent an area of 9 pure pixels (3 × 3 pixel matrix) to minimize the influence of neighboring types of coverage. The agricultural areas are located northeast of the study area (yellow circles) and comprise a plateau area, with a predominance of soybean and corn planting. The urban areas are inserted in the urban perimeters of the municipalities of Cuiabá and Várzea Grande in densely urbanized regions (red circles). Forests comprise large forest fragments and permanent preservation areas close to rivers (green circles). The areas of water bodies are inserted in the extensive system of Chacororé and Sinhá Mariana bays (blue circles), with areas of up to 64.92 km² and 11.25 km², respectively.

### 2.2. Micrometeorological Data

The flux towers continuously collected data of incident (Rgi) and reflected (Rgr) solar radiation, net radiation (Rn), soil heat flux (G), air temperature (Ta), relative humidity (RH), and wind speed (u) from 2013 to 2016. The sensors and their installed heights and the used data acquisition system in the towers are shown in Table 1.

The SEBFs and *ET* at the two flux towers were calculated using the Bowen ratio energy balance (BREB) method using the sensor listed in Table 1. This method has been widely applied and has the advantage of requiring few micrometeorological parameters while having a firm physical basis [1,39]. In addition, comparisons between estimates obtained by the BREB and the more direct eddy covariance method provide similar data, which makes the MRB an excellent method for environmental studies in remote and logistically challenging areas, such as the Cerrado-Pantanal ecotone [1,39]. The calculation of the SEBFs and *ET* is described in detail in [1].

### 2.3. Remote Sensing Data

The study was carried out with data and images obtained between 2013 and 2016 using 27 images of surface reflectance and brightness temperature from the Operational Land Imager (OLI) and the Thermal Infrared Sensor (TIRS) sensors, respectively, from Landsat 8 in path 226 and row 71, and 10 images of surface reflectance of the MOD09A1 product from the MODIS sensor on the TERRA satellite were downloaded from the EROS Science Processing Architecture (ESPA) [espa.cr.usgs.gov accessed on 25 April 2020] of the US Geological Survey (USGS).

The OLI sensor images are composed of 9 bands, with spatial resolutions of 30 m for bands 1–7 and 9, and 15 m for band 8 (panchromatic). The images from the TIRS sensor are composed of bands 10 and 11, with spatial resolution of 90 m. The temporal resolution of the Landsat 8 satellite is 16 days and the radiometric resolution is 16 bits [42]. The images of the surface reflectance without the effect of the atmosphere were processed by the Landsat Ecosystem Disturbance Adaptive Processing System (LEDAPS) hosted on the ESPA platform. LEDAPS is a complex algorithm that integrates internal sensor data (metadata) with external data (NCEP, NOAA, and NASA) to (i) transform the digital number to top of atmosphere (TOA) reflectance; (ii) detect pixels with clouds from TOA reflectance and; and (iii) calculate the corrected surface reflectance from the TOA reflectance [43]. The atmospheric correction of the surface reflectance by the LEDAPS was performed with the radioactive transfer code 6 s (Second Simulation of a Satellite Signal in the Solar Spectrum) [44], integrating (i) meteorological data from the NCEP; (ii) digital elevation models of the GCM (Global Climate Model); (iii) internal aerosol optical thickness (AOT); and (iv) ozone data collected by NASA [42,43,45]. LEDAPS also uses the digital elevation model to correct the parallax error due to the local topographic relief, as well as systematic geometric and precision corrections using surface control chips [42,43,45].

The MOD09A1 surface reflectance product of the MODIS sensor is composed of 7 bands of surface reflectance images with spatial resolution of 500 m, temporal resolution of 8 days, and radiometric resolution of 16 bits. The composition of the images allows the observation of the earth’s surface every 8 days due to high spatial coverage, low view angle, the absence or shadow of cloud, and the presence of aerosols [46]. The MOD09A1 product is equivalent to measurements at ground level with no scattering or atmospheric absorption. The product algorithm MOD09A1 corrects the effects of dispersion and absorption of gases and aerosols (atmospheric correction), as well as the adjacency effects caused by the variation of land cover, bidirectional reflectance distribution function (BRDF), and the effects of atmosphere coupling and cloud contamination. The atmospheric correction of this product was also performed by the 6 s algorithm, in which data of ozone concentration, water vapor, and aerosols were obtained from other MODIS products and auxiliary products were obtained from NASA’s Data Assimilation Office [46]. The reflectance images of the MOD09A1 product surface used in this study were obtained on the same days, or at the most ±2 days than those obtained by Landsat 8, provided there was no precipitation.

### 2.4. Surface Albedo α Models

#### 2.4.1. $\alpha_{sup}$ Using Landsat 8 (OLI)

**Figure 2:**
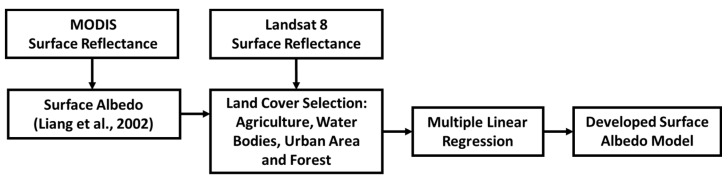
Chart flow of surface albedo model development steps from surface reflectance of the Landsat 8 OLI.

A surface albedo ($\alpha_{sup}$) model for the OLI Landsat 8 was developed in this study using a multiple linear regression of surface reflectance bands (Figure 2). The $\alpha_{sup}$ model was based on combining MOD09A1 surface albedo ($\alpha_{MODIS}$) with OLI Landsat 8 surface reflectance over different land surface cover types. The $\alpha_{MODIS}$ was used as the dependent variable and surface reflectance data from the OLI Landsat 8 were used as independent variables in the multiple linear regression equation.

The $\alpha_{MODIS}$ in this study was estimated following the approach of Liang et al. [17], as explained in Equation (1):

(1) $$\alpha_{MODIS}=0.160\rho_{1}+0.290\rho_{2}+0.243\rho_{3}+0.116\rho_{4}+0.112\rho_{5}+0.081\rho_{7}-0.0015$$

where $\rho_{1}$ to $\rho_{7}$ are the MOD09A1 surface reflectance for bands 1 to 7, respectively.

The surface reflectance images from the OLI Landsat 8 were resampled from 30 to 500 m, to have images with a spatial resolution that is consistent with those of $\alpha_{MODIS}$. The $\alpha_{sup}$ model was developed using images for Julian days 177 and 193 of the year 2013; 185, 233, and 249 of the year 2014; 217 of the year 2015; and 113, 121, and 185 of the year 2016, which provided a total of 1100 pixels obtained over agriculture, urban, forests, and water bodies areas, as shown in Figure 1. The model was validated with the image obtained on Julian day 257 of the year 2016.

#### 2.4.2. A Conventional $\alpha_{con}$ Model

Surface albedo ($\alpha_{con}$) was also estimated using a conventional model (Equation (2)) that was used in a number of studies (e.g., [47,48]). This model has been widely applied in environmental studies and in the estimation of SEBFs and *ET* algorithms, such as SEBAL [14]. It consists of a simplified radiative transfer equation that has not been evaluated in complex transition regions, such as the Cerrado-Pantanal ecotone. The surface albedo $a_{con}$ based on this model can be estimated as:

(2) $$a_{con}=\frac{\left(\alpha_{toa}-\alpha_{atm}\right)}{\tau_{oc}^{2}}$$

where $\alpha_{toa}$ is the planetary albedo; $\alpha_{atm}$ is the albedo of the atmosphere, which is generally assumed to be about [48]; and $\tau_{oc}$ is the atmospheric transmittance to global solar radiation, calculated by Equation (3) [15]:

(3) $$\tau_{oc}=0.35+0.627\exp\left[-\frac{0.00146P_{o}}{K_{t}\cos Z}-0.75\;{\left(\frac{W}{\cos Z}\right)}^{0.4}\right]$$

where $P_{o}$ is the local atmospheric pressure (kPa); $K_{t}$ is the atmospheric turbidity coefficient ($K_{t}$ = 1 if clear sky and $K_{t}$ = 0.5 if cloudy sky; we used $K_{t}=$1); and W is the precipitable water (in mm; see Equation (4)), obtained by the vapor pressure of water ($e_{a}$; in kPa):

(4) $$W=0.14\;e_{a}P_{o}+2.1$$

The albedo of the atmosphere, $\alpha_{toa}$, was calculated following Equation (5) as a linear combination of the top of atmosphere (TOA) reflectance of the OLI Landsat 8 [48], as:

(5) $$\alpha_{toa}=0.300\rho_{2}+0.277\;\rho_{3}+0.233\rho_{4}+0.143\rho_{5}+0.036\rho_{6}+0.012\rho_{7}$$

where $\rho_{2}$ to $\rho_{7}$ are top of atmosphere reflectance of bands 2 to 7 of the OLI Landsat 8.

### 2.5. Surface Temperature ($T_{s}$) Correction Models

The surface temperature ($T_{s}$) was estimated using four currently available models that include: (i) the atmospheric correction parameter calculator (ATMCORR); (ii) the single-channel (SC); (iii) the radioactive transfer equation (RTE); and (iv) the multichannel split-window (SW). These models aim to recover the radiance attenuated by atmospheric constituents in the spectral window between 10 and 13 μm.

#### 2.5.1. $T_{s_{Barsi}}$ Correction Based on ATMCORR

The ATMCORR (atmcorr.gsfc.nasa.gov, accessed on 10 August 2021) is an initiative by NASA to provide a comprehensive atmospheric correction tool for surface temperature [29,36]. ATMCORR integrates data from the National Center for Environmental Prediction (NCEP) that models the global atmospheric profile for certain dates using the well-known MODTRAN v4 code in a set of integration algorithms [29]. The atmospheric profiles generated by the NCEP integrate data from satellites and surface data to model the global atmosphere at 28 altitudes in a spatial grid of 1° × 1°. The profile data is generated every six hours with the possibility of resampling the grids. The interpolated data from the NCEP is inserted in MODTRAN v4 and the atmospheric parameters are extracted from the MODTRAN output files, adjusting the data for the moment of the satellite’s passage. Due to the robust integration of ATMCORR, this model has been widely applied in studies that demand corrected temperature [49,50]. Thus, the surface temperature obtained using the ATMCORR model as described in Barsi et al. [29], referred to in the present study as $T_{s_{Barsi}}$, was used as a reference to evaluate the $T_{s}$ as obtained by the other three temperature correction models. The $T_{s_{Barsi}}$ (K) can be calculated using Equation (6) as:

(6) $$T_{s_{Barsi}}=\frac{K_{2}}{ln\left(\frac{K_{1}}{L_{c}}+1\right)}$$

where $K_{1}$ = 607.76 W m^−2^ sr^−1^ µm^−1^ and $K_{2}$ = 1260.76 W m^−2^ sr^−1^ µm^−1^ are calibration constants of the thermal band provided by the TIRS Landsat 8 sensor; and $L_{c}$ is the radiance of a blackbody target of kinetic temperature (W m^−2^ sr^−1^ μm^−1^; see Equation (7)):

(7) $$L_{c}=\frac{L_{TOA}-L_{u}-\left(1-\varepsilon \right)L_{d}}{\tau \varepsilon }$$

$\mathrm{where}\;L_{TOA}$ is the space-reaching or *TOA* radiance measured by the TIRS (W m^−2^ sr^−1^ μm^−1^); ε is the surface emissivity over TIRS band calculated by Equation (8) [51] and the parameters obtained by ATMCORR; $L_{u}$ is the upwelling or atmospheric path radiance (W m^−2^ sr^−1^ μm^−1^); $L_{d}$ is the downwelling or sky radiance (W m^−2^ sr^−1^ μm^−1^); and τ is the thermal atmospheric transmission.

(8) $$\varepsilon =\varepsilon_{s}\;\left(1-FVC\right)+\varepsilon_{v}FVC$$

where $\varepsilon_{s}$ and $\varepsilon_{v}$ denote bare soil and vegetation emissivity, respectively, over the TIR band; and *FVC* is the fraction of vegetation cover (Equation (9)):

(9) $$FVC={\left(\frac{NDVI-NDVI_{min}}{NDVI_{max}-NDVI_{min}}\right)}^{2}$$

where NDVI is the normalized difference vegetation index; and $NDVI_{min}$ and $NDVI_{max}$ are the minimum and maximum NDVI, respectively, extracted from the *NDVI* histogram.

#### 2.5.2. $T_{s_{SC}}$ Correction Based on the Single-Channel (SC) Model

The single-channel (SC) model consists of the correction of surface temperature ($T_{s_{SC}}$; see Equation (10)) based on correction functions γ, ψ, and δ that can be estimated by the parameters $L_{u}$, $L_{d}$, and τ [34]. The SC model can be applied to any of the bands in the Landsat 8 TIRS. This study used band 10 to correct $T_{s}$ [referred to as $T_{s_{SC}}$]:

(10) $$T_{s_{SC}}=\gamma \left[\frac{1}{\varepsilon }\;\left(\psi_{1}L_{toa}+\psi_{2}\right)+\psi_{3}\right]+\delta$$

where $\psi_{1}$, $\psi_{2}$, and $\psi_{3}$ are atmospheric correction functions, calculated by Equations (11)–(13) from parameters obtained from ATMCORR; and γ and δ are functions of $L_{toa}$, brightness temperature ($T_{b}$; K), and $b_{\gamma }$, which is equal to $K_{1}$ [51]:

(11) $$\psi_{1}=\frac{1}{\tau }$$

(12) $$\psi_{2}=-L_{d}$$

(13) $$\psi_{3}=L_{d}$$

(14) $$\gamma \approx \frac{T_{b}{}^{2}}{b_{\gamma }L_{toa}}$$

(15) $$\delta \approx Tb-\frac{Tb^{2}}{b_{\gamma }}$$

#### 2.5.3. $T_{s_{RTE}}$ Correction Based on the RTE Model

The corrected $T_{s}$ using the radiative transfer equation is referred to in this article as $T_{s_{RTE}}$ (K), and was calculated following Equation (16) based on the $L_{toa}$ and the parameters obtained by ATMCORR [51]:

(16) $$T_{s_{RTE}}=\frac{C_{2}}{\lambda \cdot ln\left(\frac{C_{1}}{\lambda^{5}\cdot L_{c}}+1\right)}=\frac{C_{2}}{\lambda \cdot ln\left\{\frac{C_{1}}{\lambda^{5}\cdot \left[\frac{L_{toa}-L_{u}-\tau \left(1-3\right)L_{d}}{\tau \varepsilon }\right]}+1\right\}}$$

where $C_{1}$ = 1.19104 × 10^8^ W μm^4^ m^−2^ sr^−1^ and $C_{2}$ = 14387.7 μm K are constant; and λ is the effective wavelength of the band.

#### 2.5.4. $T_{s_{SW}}$ Correction Based on the Split-Window (SW) Model

The split-window surface temperature correction model is one of the simplest techniques, in which the radiation attenuation by atmospheric absorption is proportional to the difference in radiance measured simultaneously by the two thermal bands [28,34]. The surface temperature ($T_{s_{SW}}$; K) based on the SW model can be calculated as:

(17) $$T_{s_{SW}}=T_{b10}+c_{1}\;\left(T_{b10}-T_{b11}\right)+c_{2}{\left(T_{b10}-T_{b11}\right)}^{2}+c_{0}+\left(c_{3}+c4\;w\right)\left(1-\varepsilon \right)+\left(c_{5}+c_{6}w\right)\Delta \varepsilon$$

where $T_{b10}$ and $T_{b11}$ are the brightness temperature of bands 10 and 11 (K) of TIRS; $c_{x}$ is constant with the following values $c_{0}$ = −0.268, $c_{1}$ = 1.378, $c_{2}$ = 0.183, $c_{3}$ = 54.30, $c_{4}$ = −2.238, $c_{5}$ = −129.20, and $c_{6}$ = 16.40 [34]; Δε is the difference in emissivity of the thermal bands 10 and 11 of TIRS; and w is the water vapor concentration (g cm^−2^) calculated by Equation (18) [52].

### 2.6. Estimation of SEBFs and ET Using SEBAL

**Figure 3:**
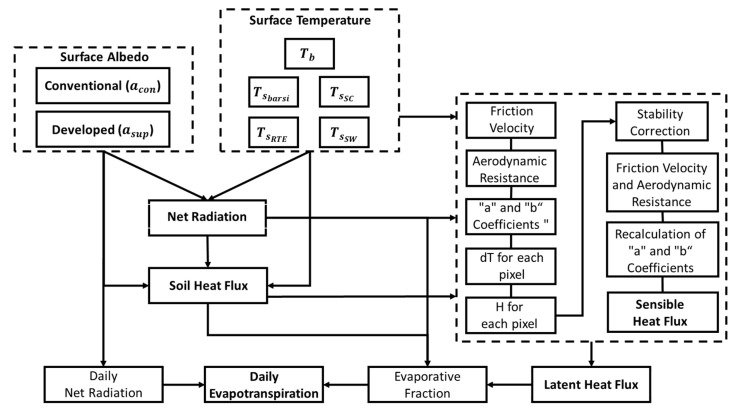
Flowchart of the processing steps of the SEBAL algorithm.

The SEBAL algorithm was processed according to the flow chart shown in Figure 3. It was proposed to estimate the daily evapotranspiration (*ET*) from the instantaneous latent heat flux (LE; W m^−2^) obtained as a residue of the energy balance equation (Equation (18)):

(18) LE=Rn−G−H

where Rn is the net radiation (W m^−2^); G is the soil heat flux (W m^−2^); and H is the sensible heat flux (W m^−2^).

The Rn (Equation (19)) represents the balance of short-wave and long-wave radiation on the surface:

(19) $$Rn=R_{s↓}\;\left(1-\alpha \;\right)+R_{L↓}-R_{L↑}-\left(1-\;\varepsilon \right)R_{L↓}$$

where $R_{s↓}$ is the measured incident solar radiation (W m^−2^); α is the surface albedo; $R_{L↓}$ is the long-wave radiation emitted by the atmosphere in the direction of the surface (W m^−2^); $R_{L↑}$ is the long-wave radiation emitted by the surface to the atmosphere (W m^−2^); and ε is the surface emissivity. The $R_{L↑}$ and $R_{L↓}$ were calculated by Equations (20) and (21):

(20) $$R_{L↑}=\varepsilon_{sup}.\sigma .T_{s}^{4}$$

(21) $$R_{L↓}=\varepsilon_{atm}.\sigma .T_{a}^{4}$$

where $\varepsilon_{sup}$ and $\varepsilon_{atm}$ are the surface and atmosphere emissivity; σ are the Stefan–Boltzmann constant (σ = 5.67.10^−8^ W m^−2^ K^−4^); $T_{s}$ is the surface temperature (K); and $T_{a}$ is the air temperature (K). The $R_{L↑}$ was calculated using the surface temperature calculated by the models described in item 2.6.

The G was calculated by Equation (22) [12]:

(22) $$G=Rn\left[T_{s}\left(0.0038+0.0074\alpha_{sup}\right)\;\left(1-0.98NDVI^{4}\right)\right]$$

where $T_{s}$ is the surface temperature (K) calculated by the different models described in Section 2.6; $\alpha_{sup}$ is surface albedo calculated by the models described in Section 2.4 and Section 2.5; NDVI is the normalized difference vegetation index; and Rn is the net radiation calculated by the different $T_{s}$ models described in Section 2.6 and $\alpha_{sup}$ described in Section 2.4 and Section 2.5.

H is the central variable in the SEBAL algorithm and estimated by the classic aerodynamic (Equation (23)) [8]:

(23) $$H=\rho c_{p}\frac{\left(dT\right)}{r_{ah}}$$

where ρ is the specific air mass (kg m^−3^); $c_{p}$ is the specific heat of air at a constant pressure (1004 J kg^−1^ K^−1^); dT is the temperature difference near the surface (K); and $r_{ah}$ is the aerodynamic resistance to the transport of sensible heat flux (s m^−1^) between two heights ($z_{1}$ = 0.1 m and $z_{2}$ = 2.0 m). The $r_{ah}$ is obtained as a function of the friction speed after an iterative correction process based on atmospheric stability functions [8].

The dT was calculated from a linear relationship with the $T_{s}$ (Equation (24)), and the values of the coefficients “*a*” and “*b*” were obtained using information from two “anchor” pixels [8]:

(24) $$dt=a+bT_{s}$$

In SEBAL, the “anchor” pixels represent conditions of hydrological extremes, in which “cold” represents surfaces with H close to zero and “hot” surfaces with LE close to zero. In general, the cold pixel can be represented by a body of water or a well-irrigated crop, and the hot pixel can be represented by a severe surface water restriction, such as exposed soils [8].

In non-agricultural environments, as those of concern in this study, the conditions for choosing the cold pixel may not be properly satisfied, restricting the choice of the cold pixel in areas of native forest. In this study, an approach similar to that used in METRIC was used, using the values of Rn and G of the cold pixel of a known surface and the actual evapotranspiration (ETr) from an estimate reference evapotranspiration (ETo), with local weather station data and the cultivation coefficient (Kc) of the cold pixel surface [15]. Then, the ETr was converted to LE to obtain the H of cold pixel. Thus, it was possible to find the coefficients of Equation (24) and solve the dT by the system formed by Equations (23) and (24) in an iterative process.

After obtaining the LE of each pixel by Equation (18), the daily evapotranspiration (ET; mm d^−1^) of each pixel was calculated by Equation (25), from the instantaneous evaporative fraction ($FE_{i}$; see Equation (26)) and daily Rn ($Rn_{24h}$; W m^−2^) of each pixel and the latent heat of vaporization of water (λ; kg m^−3^) [12]:

(25) $$ET=\frac{\left(86400\times FE_{i}\times Rn_{24h}\right)}{\lambda }$$

(26) $$FE_{i}=\left(\frac{LE}{Rn-G}\right)$$

### 2.7. Evaluation Approach and Performance Indicators

This study followed four steps to evaluate the effects of surface albedo and temperature models on SEBFs and *ET* that include:

1. Developing a surface albedo model by combining MODIS and Landsat 8 dataset. A subset of the data was used for model development and the remaining was used to evaluate the model performance over different land cover types. In this analysis, the MODIS surface albedo by Liang et al. [17] was assumed to be as a reference against which to compare the developed and existing models.
2. Comparing the performance of the of the developed surface albedo model with the currently used conventional model.
3. Retrieving and evaluating land surface temperature based on four different methods. In this analysis, the model by Barsi, et al. [29] was assumed to be the reference against which to compare other retrieval methods. The comparison between the different retrieval methods was conducted over the sample sites.
4. Evaluating the combined effects of the surface albedo models and the brightness temperature and temperature retrieval methods on SEBFs and *ET*. Since both variables (i.e., α and $T_{s}$) are used in SEBAL model to estimate SEBFs and *ET*, a set of combinations of the two variables were developed as shown in Table 2 to identify these effects.

The averages of all variables were calculated with a confidence interval (CI) of ±95% using bootstrapping of 1000 iterations of random resamples with substitution [54]. The accuracy of surface albedo models analyzed in this study as well as the estimated SEBFs and *ET* were assessed using the Willmott coefficient (d; see Equation (27)), the root mean square error (RMSE; see Equation (28)), the mean absolute error (MAE; see Equation (29)), the mean absolute percentage error (MAPE; see Equation (30)), and the Pearson’s correlation coefficient (r):

(27) $$d=\left[\frac{\sum_{i=1}^{n}{\left(E_{i}-O_{i}\right)}^{2}}{\sum_{i=1}^{n}{\left(\left|E_{i}-\bar{O}\right|+\left|O_{i}-\bar{O}\right|\right)}^{2}}\right]$$

(28) $$RMSE={\left(\frac{\sum_{i}^{N}{\left(E_{i}-O_{i}\right)}^{2}}{n}\right)}^{\frac{1}{2}}$$

(29) $$MAE=\frac{1}{n}\overset{n}{\underset{i=1}{\sum }}\left|E_{i}-O_{i}\right|\;$$

(30) $$MAPE=\frac{100}{n}\;\overset{n}{\underset{i=1}{\sum }}\left|\frac{E_{i}-O_{i}}{O_{i}}\right|$$

where $E_{i}$ are the estimated values; $O_{i}$ are the observed values; $\bar{O}$ is the average of the observed values; and n are sample numbers. In the case of surface albedo models, the observed values were based on MODIS surface albedo ($\alpha_{MODIS}$), while in the case of SEBFs and *ET*, the observed values were obtained from the ground measurements at the flux sites FMI and BPD. The Willmott coefficient relates the model’s performance based on the distance between estimated and observed values, with values ranging from zero (without agreement) to 1 (perfect agreement). The RMSE indicates how much the model fails to estimate the variability of the measurements around the mean value, as well as the variation of the estimated ones around the observed values [55]. The MAE indicates the absolute mean distance (deviation) and the MAPE indicates the average percentage of the difference between the estimated and observed values. The lowest value of RMSE, MAE, and MAPE is 0, which means that there is complete agreement between the estimated and observed values.

## 3. Results

### 3.1. Surface Albedo Model Based on the OLI Landsat 8

The surface albedo ($a_{sup}$) model developed in this analysis based on the surface reflectance of the OLI Landsat 8 is shown in Equation (32):

(31) $$a_{sup}=0.4739\rho_{2}-0.4372\rho_{3}+0.1652\rho_{4}+0.2831\rho_{5}+0.1072\rho_{6}+0.1029\rho_{7}+0.0366$$

where $\rho_{2}\;\mathrm{to}\;\rho_{7}$ represent the surface reflectance of the OLI Landsat 8 for bands 1 to 7, respectively.

A comparison of the surface albedo between $a_{MODIS}$ and $a_{sup}$ as well as between $a_{MODIS}$ and $a_{con}$ indicated that $a_{sup}$ performed better than $a_{con}$, as shown in Table 3. The summary of the comparison shown in Table 2 was based on surface albedo values from all selected sites. The average of $a_{sup}$ was not significantly different from that of $a_{MODIS}$, while the average of $a_{con}$ was 49% higher than the that of $a_{sup}$ (Table 3). The RMSE of $a_{sup}$ was 5.6-fold lower and the Willmott and correlation coefficients were approximately 2-fold higher for α*_sup_* than $a_{con}$.

Regarding the performance of $a_{sup}$ over the different land use types, it appears that $a_{sup}$ had better performance than $a_{con}$ over the different sampled land uses. The averages $a_{sup}$ and $a_{MODIS}$ were similar in pasture and urban areas, and they were close in the forest and water bodies, while the means of $a_{con}$ were from 36% to 64% higher than $a_{MODIS}$ (Table 4).

### 3.2. $T_{s}$ Retreival Models

**Figure 4:**
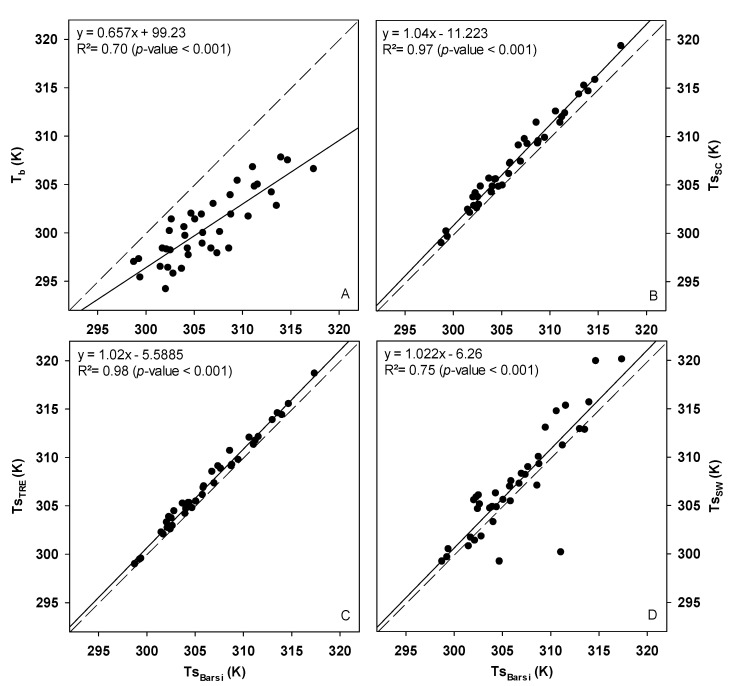
Relation of (**A**) the surface temperature corrected by the Barsi model ($T_{s_{barsi}}$; K) and brightness temperature ($T_{b}$); (**B**) the surface temperature corrected by the single-channel model ($T_{s_{SC}}$; K); (**C**) the surface temperature corrected by the radiative transfer equation model ($T_{s_{RTE}}$; K); and (**D**) the surface temperature corrected by the split-window model ($T_{s_{SW}}$; K).

Based on a comparison with $T_{s_{barsi}}$, the results indicated that $T_{s_{SC}}$ and $T_{s_{RTE}}$ had much lower discrepancies based on the obtained *MAE*, *MAPE*, and *RMSE*, and higher agreement based on the Willmott coefficient (d) and Pearson correlation (r), compared to $T_{s_{SW}}$ and $T_{b}$ (Figure 4 and Table 5). The averages of $T_{s_{barsi}}$, $T_{s_{SC}}$, $T_{s_{RTE}}$, and $T_{s_{SW}}$ were not significantly different; however, $T_{b}$ was lower than $T_{s_{barsi}}$ by about 2%. The largest correction error was observed when comparing $T_{b}$ with $T_{s_{barsi}}$, while $T_{s_{RTE}}$ had the least errors compared to $T_{s_{barsi}}$. The surface temperatures ($T_{s}$) corrected by the different models had MAE and RMSE up to 86% lower than the $T_{b}$.

### 3.3. SEBFs and ET Estimates Based on α and $T_{s}$ Combinations

A summary of the comparison between estimated and measured *Rn* based on all model combinations (Table 2 over both flux towers, i.e., FMI and PBE) is shown in Table 6. A comparison of *Rn* estimates with measurements over each individual tower is shown in the Supplementary Material. The averages of estimated Rn based on the different α and $T_{s}$ combinations (Table 2) of $a_{sup}$ with all $T_{s}$ as well as the combination of $a_{con}$ with $T_{b}$ did not show a significant difference from the values measured at the flux towers, but the average of estimated Rn based on the combination of $a_{con}$ and all $T_{s}$ were 15% lower than the measured Rn (Table 6). The estimated Rn with the combination of $a_{sup}$ and $T_{b}$ had the lowest errors and the highest Willmott’s d and r, while the highest errors and lowest coefficient d and r were observed with the combination of $a_{con}$ and $T_{s_{SW}}$ (Table 6).

Unlike Rn, the averages of estimated G based on $a_{sup}$ with all $T_{s}$ retrieval methods, including $T_{b}$, did not differ between each other, but were between 35% and 54% higher than the measured G (Table 7). The values of d and r changed significantly, but the errors in estimated G with $T_{b}$ were 18% less than with $T_{s}$.

The average of estimated H based on all combinations of $a_{sup}$ and $T_{s}$ as well as the combination of $a_{con}$ with $T_{b}$ did not show a significant difference from those of the measured values, while the averages of estimated H based on $a_{con}$ and the different $T_{s}$ were between 26–35% lower than those of the measured values (Table 8). The *MAE* and *RMSE* in estimating H with $a_{sup}$ were between 7–47% less than those based on $a_{con}$. The estimated H with the combination of $a_{sup}$ and $T_{s_{barsi}}$ had the smallest *MAE*, *MAPE*, and *RMSE*, while the largest *MAE*, *MAPE*, and *RMSE*, and smallest d and r were obtained with the combination of $a_{con}$ and $T_{s_{SW}}$.

Opposed to what was observed with the Rn, G and H, there was no difference in the averages of estimated LE and ET based on the different combinations of $a_{sup}$ and $T_{s}$ (Table 9 and Table 10). It should be noted that the *MAE*, *MAPE*, and *RMSE* in LE and ET estimates with $a_{sup}$ were on average 28% and 20% lower, respectively, and the coefficients were slightly higher than those estimated with $a_{con}$, with emphasis on the combination of $a_{sup}$ and $T_{s_{barsi}}$.

## 4. Discussion

### 4.1. Surface Albedo Models Performance

The surface albedo model ($a_{sup}$) developed in this study performed well compared to the conventional one ($a_{con}$). The RMSE based on $a_{sup}$ was less than 0.03 required by climate forecasting models [18] and within the range of 0.01–0.02 found in previous studies [18,56]. The largest discrepancies shown by $a_{con}$ as indicated in the reported *MAE* and *RMSE* can be due a number of factors that include (i) the broad spectrum or broadband transmittance being inadequate for the atmospheric correction of the composition of discrete bands; (ii) not considering the differences in atmospheric transmissivity for each band; and (iii) the non-correspondence of the narrow and wide bands with solar radiation on the surface [14].

The $a_{sup}$ in the four land types was within the range found in other studies. The $a_{sup}$ over agricultural areas ranged from 0.14 to 0.18 [56,57]; from 0.15 to 0.20 over urban areas due to the complexity of mixtures of built-up area and vegetation in backyards and streets [58]; from 0.11 to 0.13 over the Cerrado forest [57,59]; and from 0.05 to 0.07 over water bodies depending on the composition of the water [58,59]. The $a_{con}$ of water bodies was greater than 0.10, which is above the values obtained in the lakes of the region [59].

### 4.2. Evaluation of $T_{s}$ Retrieval Models

In general, the difference between $T_{b}$ and $T_{s}$ varies between 1 and 5 K in the 10–12 μm spectral region, subject to the influence of atmospheric conditions and surface emissivity [60]. For mathematical convenience, the TOA thermal radiation is generally expressed in terms of $T_{b}$ with an emissivity of 1.0 [61]. The TOA radiance is the result of radiation emitted by the Earth’s surface, upward radiation emitted by the atmosphere, and downward radiation emitted by the atmosphere [62]. The TOA radiation is mostly attenuated by water vapor and, to a lesser extent, by trace gases and aerosols [63,64].

In this study, $T_{s_{barsi}}$ was used as a reference to validate $T_{b}$, $T_{s_{SC}}$, $T_{s_{RTE}}$, and $T_{s_{SW}}$, because previous studies indicated $T_{s_{barsi}}$ based on Landsat had relatively low *MAE* and *RMSE* values low (ranging between 0.2 and 2.5 K) when compared to the field measurements of $T_{s}$ that were taken over different land surface types represented by the Surface Radiation Budget (SURFRAD) Network (https://gml.noaa.gov/grad/surfrad/ accessed on 10 August 2021) during different atmospheric profiles [65,66,67,68]. Furthermore, the improved performance of $T_{s_{barsi}}$ is associated with the robustness of the MODTRAN algorithm and the integration of atmospheric data from the NCEP to generate the atmospheric correction parameters ($L_{u}$, $L_{d}$, and τ) [36]. These parameters estimated by ATMCORR were also used in the other models, which may justify the good relationship between the $T_{s}$ estimated by the $T_{s_{SC}}$, $T_{s_{RTE}}$, and $T_{s_{SW}}$.

The good relationships between $T_{s_{SC}}$, $T_{s_{RTE}}$, and $T_{s_{SW}}$ with $T_{s_{barsi}}$ obtained in this study agreed with other validation and simulation studies, which indicated that the *MAE* and *RMSE* obtained in this study are within those limits reported in the literature. The typical *MAE* and *RMSE* of $T_{s_{SC}}$ and $T_{s_{RTE}}$ vary between 1 and 3 K [31,69], and the $T_{s_{SW}}$ is around 1.5 K [33]. Using low spatial resolution data, $T_{s_{SC}}$ and $T_{s_{RTE}}$ presented *MAE* and *RMSE* from 1.6 to 2.4 K [70], and $T_{s_{SW}}$ from 1.5 to 2.9 K [71].

The good agreement of $T_{s_{RTE}}$ with $T_{s_{barsi}}$ maybe due to both models using the radiative transfer equation of Planck’s inverse equation [29,30,35,51]. The main difference of $T_{s_{RTE}}$ and $T_{s_{barsi}}$ is on the conversion of thermal radiance into $T_{s}$, since $T_{s_{RTE}}$ is converted by the inverted Plank equation and $T_{s_{barsi}}$ by a specific Planck curve equation with calibration constants determined for the TIRS Landsat 8 [35,36]. $T_{s_{RTE}}$ has been widely used in studies of water bodies with an accuracy of around 0.2 K and in studies of terrestrial bodies with errors of up to 2 K [35,72].

The RMSE of $T_{s_{SC}}$ around 1.3 K showed its good agreement with $T_{s_{barsi}}$, at the lower limit of the range from 1.2 to 2 K obtained under different conditions of atmospheric water vapor [30,34]. The biggest errors of $T_{s_{SW}}$ can be attributed to the model being multichannel, which introduces greater noise if using only one thermal channel [28,34,73]. However, $T_{s_{SW}}$ is obtained by combining thermal bands with defined coefficients, considering different emissivity for each band and requiring only knowledge of the atmospheric water vapor [28,34].

### 4.3. The Effects of α and $T_{s}$ Retreival Models on SEBFs and ET

In general, RMSE of Rn is typically found to be between 20 and 80 W m^−2^ with different orbital sensors (TM Landsat 5, TM+ Landsat 7, and MODIS) [59,74,75,76,77,78,79,80]. The RMSE obtained in this study were close to those reported by [59] over the Cerrado zone and by [10] on the Cerrado-Pantanal transitional zone in Brazil, which highlight the relatively acceptable accuracy of estimated Rn obtained based on all combinations. The better performance of the Rn estimated with the $T_{b}$ maybe due to the shortwave and longwave radiation balance [10]. The $a_{sup}$ can be overestimated by up to 15%, which leads to an underestimation of Rn [11,81], while $T_{b}$ is generally lower than $T_{s}$, leading to an underestimation of long-wave radiation emitted by the surface ($R_{L↑}$), which therefore leads to overestimation of Rn. Despite the better performance of Rn with $T_{b}$, the MAPE of Rn estimated with $a_{sup}$ and all $T_{s}$ were less than 2%, and the *RMSE* less than 20 W m^−2^. In addition, the difference in MAE and RMSE of the estimated Rn with all $T_{s}$ and the same surface albedo model was less than 5 W m^−2^ and MAPE less than 1%.

The obtained MAE and RMSE values of G were within the range of 15–32 W m^−2^, which was similar to those obtained in other studies [82,83]. The low performance of G has been reported in other studies with different land uses [82,83,84]. Probably, the low performance of the G estimate is due to the low sensitivity of the model to the high spatial complexity of the study area. G tends not to have a high impact on the SEB and ET of densely vegetated surface, due to the lesser part of the available energy used to heat the soil, but G tends to impact the SEB and ET of surfaces with low vegetation cover, as the pastures and some natural grasslands in Cerrado and Pantanal [13,82,85].

The MAE and RMSE of H estimated based on all combinations with $a_{sup}$ and the combination of $a_{con}$ with $T_{b}$ were less than the 50 W m^−2^ that was reported by [6,78,82,86]. Estimates of H with $T_{s_{barsi}}$, $T_{s_{SC}}$, and $T_{s_{RTE}}$ were on average 3% lower than that with $T_{b}$, indicating that the differences between $T_{s}$ and $T_{b}$ do not significantly impact H. This is because the internal calibration process of SEBAL alleviates impacts of low $T_{b}$ values [11]. The estimation of H by SEBAL is a function of the linear relationship “$dTs=a+bT_{s}$”, using two extreme pixels to calculate the constants “a” and “b” [8,15]. The initial value of these constants is obtained from meteorological information, satellite estimates (Rn−G; SAVI), and the operator’s choice (anchor pixels), and these constants are adjusted by iterations [11,15,87]. The estimation of these constants by numerical iterations eliminates the effects of the negative bias of $T_{b}$ and transmits the calibration effect for all other pixels in proportion to the inserted $T_{s}$. Therefore, the differences between $T_{s}$ and $T_{b}$ tend not to significantly affect SEBF estimates [11,16,87].

The MAE and RMSE of LE estimates were within the range of 30–70 W m^−2^ found in previous studies based on measurements with flux towers and lysimeters, and the MAPE was less than 20% [78,82,84,86,88]. The MAE and RMSE of the ET estimates were also within the range of 0.3 mm to 0.6 mm day^−1^, and the MAPE within the range of 8% to 20% found in other studies [6,78,89,90,91]. In this study, SEBAL was applied in areas with grasses and shrubland typical of the Cerrado-Pantanal transition region under different natural water conditions and obtained errors between 11% and 12.5%, which represented absolute errors of ET less than 0.35 mm day^−1^.

The slight difference between the *MAE*, *MAPE*, and *RMSE* and the correlation and Willmott coefficients of the LE and ET estimated with $T_{b}$ and $T_{s}$ shows that the recovery of $T_{s}$ by the models does not significantly impact the estimation of these parameters. This effect was also observed in studies by [11] and [16]. This reinforces that the internal calibration of SEBAL keeps the “$dTs=a+bT_{s}$” stable and minimizes the impacts of the insertion of $T_{s}$ in the LE and ET estimates, since the $T_{s}$ of the anchor pixels represent the extreme conditions of water availability, regardless of the removal of the effect of the atmosphere and the emissivity of the surface on the thermal band [11,87]. In contrast, LE and ET performed better with $a_{sup}$ instead of $a_{con}$. A similar result was also observed in the work of [11], which proposed an internal SEBAL calibration to remove the effect of the atmosphere in each band of the Landsat 5 sensor, whose estimate of ET with $a_{con}$ introduced random errors of ±1 mm day^−1^.

## 5. Conclusions

In this study, a model of surface albedo ($a_{sup}$) with the OLI Landsat 8 surface reflectance was developed. Surface temperature ($T_{s}$) was recovered by different models from the brightness temperature ($T_{b}$). The performance of surface energy balance fluxes (SEBF) and evapotranspiration (ET) estimates, based on different combinations of surface albedo and temperature models, was evaluated against ground-based observations of SEBF. The $a_{sup}$ model performed better than a conventional surface albedo model ($a_{con}$) as it provided lower *MAE*, *MAPE*, and *RMSE* and higher Willmott coefficients (d) and Pearson correlation (r) when compared with surface albedo data based on MODIS ($a_{MODIS}$). In addition, average values of $a_{sup}$ were similar to those found by $a_{MODIS}$, while those of $a_{con}$ were about 36–64% higher than $a_{MODIS}$. Additionally, $a_{con}$ showed some limitations over water bodies. Minimizing these errors in spatially complex areas, such as the Cerrado-Pantanal transition, is important for accurate estimates of SEBFs and *ET*.

The retrieval of surface temperature ($T_{s}$) by the different models combined with $a_{con}$ significantly influenced estimates of the net radiation (Rn) and the sensible heat flux (H). Estimates of the Rn were on average 15% lower and those of H, which were about 26–35% lower than the measured Rn and H, respectively. However, estimates of Rn and H based on the combination of $T_{s}$ with $a_{sup}$ were not significantly different from those measured. Moreover, the averages of latent heat flux (LE) and evapotranspiration (ET) were also not significantly different from those measured based on all combinations.

The determination of the $a_{sup}$ model, with the OLI Landsat 8 surface reflectance for the studied Cerrado-Pantanal transition region, improved the performance of SEBAL in estimating the Rn, H, LE, and ET, when combined with both $T_{s}$ and $T_{b}$. SEBFs and ET estimated by SEBAL with $a_{sup}$ had lower errors (i.e., *RMSE*) and higher agreement and correlation coefficients d and r. It is noteworthy that the SEBFs and ET estimated by the combination $a_{sup}$ and $T_{s_{barsi}}$ presented the best performance. The combination of $a_{con}$ and $T_{s_{SW}}$ worked well to estimate *ET* over the mixed shrub–grass site of the PBE, while combination of $a_{sup}$ and $T_{b}$ worked well to estimate *ET* over the grassland site of the FMI. The evaluation conducted in this analysis over the spatially complex gradient of natural ecosystems in southern Brazil provided a robust test of the performance of these surface albedo and temperature algorithms and can help to guide future studies on the use of appropriate models for the estimation of SEBFs and *ET* over other regions with similar complex environments.

## Figures and Tables

**Table 1:** Description of the equipment used to measure incident solar radiation (Rgi), reflected solar radiation (Rgi), net radiation (Rn), soil heat flux (G), air temperature (Ta), relative humidity (RH), wind speed (u), datalogger, and their respective heights in the Fazenda Miranda (FMI) and Baía das Pedras (BPE) flux towers.

Variable	Equipment Description	Installation Height from the Ground (m)	
		FMI	BPE
Rgi/Rgr	LI200X, LI-COR, Lincoln, NE, USA	5	20
Rn	NRLITE, Kipp & Zonen, Delft, The Netherlands	5	20
G	HFP01, Hukseflux BV, Delft, The Netherlands	−0.05	−0.05
Ta/RH	HMP-45AC, Vaisala Inc., Woburn, USA	5–18	22–31
u	014A, Met One, Grants Pass, USA	5	22
Datalogger	CR1000, Campbell Scientific, Inc., Logan, USA		

**Table 2:** Summary of model combinations used to evaluate the effects of the surface albedo estimated by the conventional model ($a_{con}$) and the model developed in this study ($a_{sup}$) and the surface brightness temperature ($T_{b}$), and the surface temperature retrieved by the Barsi model ($T_{s_{barsi}}$), the single-channel model ($T_{s_{SC}}$), the radiative transfer equation model ($T_{s_{RTE}}$), and the split-window model ($T_{s_{SW}}$) on surface energy balance and evapotranspiration.

Combinations of α $\mathrm{and}\;T_{s}\mathrm{Models}\;\mathrm{Used}\;\mathrm{to}\;\mathrm{Evaluate}\;\mathrm{SEBFs}\;\mathrm{and}\;\mathrm{ET}$				Evaluation Sites
Surface Albedo (α)	Source	$\mathrm{Surface}\;\mathrm{Temperature}\;(T_{s})\;\mathrm{Retrieval}$	Source	
$a_{con}$		$T_{b}$	USGS, [53]	FMI (Mixed woodland–grassland) and BPE (Seasonal flooded large shrubs)
		$T_{s_{barsi}}$	Barsi et al. [29]	
	Silva et al. [48]	$T_{s_{SC}}$	Jimenez-Munoz et al. [34]	
		$T_{s_{RTE}}$	Jimenez-Munoz et al. [51]	
		$T_{s_{SW}}$	Jimenez-Munoz et al. [34]	
$a_{sup}$		$T_{b}$	USGS, [53]	FMI (Mixed woodland–grassland) and BPE (Seasonal flooded large shrubs)
		$T_{s_{barsi}}$	Barsi et al. [29]	
	This study	$T_{s_{SC}}$	Jimenez-Munoz et al. [34]	
		$T_{s_{RTE}}$	Jimenez-Munoz et al. [51]	
		$T_{s_{SW}}$	Jimenez-Munoz et al. [34]	

**Table 3:** Average (±95% confidence interval) of the surface albedo estimated by MODIS ($a_{MODIS}$) used as reference values, and the average (±95% confidence interval), mean absolute error (*MAE*), mean absolute percent error (*MAPE*, %), root mean square error (*RMSE*), Willmott coefficient (d), and Pearson correlation coefficient (r) of the surface albedo estimated by the model developed in this study ($a_{sup}$) and the surface albedo estimated by the conventional model ($a_{con}$). Values with (***) indicate *p*-value < 0.001. All units are dimensionless.

Models	Average ± IC	*MAE*	*MAPE*	*RMSE*	*d*	*r*
$a_{MODIS}$ *	0.159 ± 0.005					
$a_{sup}$	0.155 ± 0.004	0.011	7.12	0.014	0.89	0.79 ***
$a_{con}$	0.232 ± 0.009	0.072	46.12	0.079	0.40	0.64 ***

* The $a_{MODIS}$ was used as a reference to evaluate other surface albedo methods.

**Table 4:** Average (±95% confidence interval) of the surface albedo estimated by MODIS ($a_{MODIS}$), used as reference values, surface albedo estimated by the model developed in this study ($a_{sup}$) and surface albedo estimated by the conventional model ($a_{con}$) in agriculture, urban area, forest, and water bodies on the study area. All units are dimensionless.

Models	Average ± IC Surface Albedo Values over Different Land Use Types			
	Agriculture	Urban Area	Forest	Water Bodies
$a_{MODIS}$	0.179 ± 0.004	0.168 ± 0.004	0.125 ± 0.001	0.08 ± 0.003
$a_{sup}$	0.173 ± 0.003	0.162 ± 0.006	0.130 ± 0.002	0.07 ± 0.002
$a_{con}$	0.244 ± 0.007	0.275 ± 0.030	0.178 ± 0.003	0.18 ± 0.004

**Table 5:** Average (±95% confidence interval) of the surface temperature corrected by the Barsi model ($T_{s_{barsi}}$; K), used as reference values, and average (±95% confidence interval), mean absolute error (*MAE*), mean absolute percent error (*MAPE*), root mean square error (*RMSE*), Willmott coefficient (d) and Pearson correlation coefficient (r) of the surface temperature corrected by the single-channel model ($T_{s_{SC}}$; K), the radiative transfer equation model ($T_{s_{RTE}}$; K), and the split-window model ($T_{s_{SW}}$; K). Values with (***) indicate *p*-value < 0.001.

Models	Average ± IC	*MAE*	*MAPE*	*RMSE*	d	r
	K	K	%	K		
$T_{s_{barsi}}$ *	306.3 ± 1.45					
$T_{b}$	300.5 ± 1.1	5.76	1.87	6.27	0.63	0.83 ***
$T_{s_{SC}}$	307.5 ± 1.5	1.06	0.34	1.28	0.98	0.98 ***
$T_{s_{RTE}}$	307.1 ± 1.5	0.78	0.25	0.95	0.98	0.99 ***
$T_{s_{SW}}$	307.2 ± 1.75	1.89	0.61	2.78	0.91	0.86 ***

* The $T_{s_{barsi}}$ was used as a reference to evaluate other surface temperature retrieval methods.

**Table 6:** Average (±95% confidence interval) of the measured net radiation (Rn; W m^−2^) in the flux towers, and the average (±95% confidence interval), mean absolute error (*MAE*), mean absolute percent error (*MAPE*), root mean square error (*RMSE*), Willmott coefficient (d), and Pearson correlation coefficient (r) of the estimated net radiation using the conventional ($a_{con}$) and parameterized ($a_{sup}$) surface albedo models combined with brightness temperature ($T_{b}$) and the surface temperature corrected by the Barsi model ($T_{s_{barsi}}$), the single-channel model ($T_{s_{SC}}$), the radiative transfer equation model ($T_{s_{RTE}}$), and the split-window model ($T_{s_{SW}}$). Values with (***) indicate *p*-value < 0.001.

		Average ± IC	*MAE*	*MAPE*	*RMSE*	d	r
		W m^−2^	W m^−2^	%	W m^−2^		
Measured Rn		510.1 ± 30.0					
**Model Combination**							
$a_{con}$	$T_{b}$	475.6 ± 22.0	33.41	6.24	43.64	0.92	0.94 ***
	$T_{s_{barsi}}$	428.3 ± 22.0	66.00	12.66	77.98	0.79	0.88 ***
	$T_{s_{SC}}$	432.1 ± 23.0	72.59	13.94	85.60	0.76	0.85 ***
	$T_{s_{RTE}}$	434.2 ± 23.0	70.83	13.60	83.54	0.77	0.86 ***
	$T_{s_{SW}}$	432.4 ± 23.0	72.63	13.90	86.14	0.75	0.83 ***
$a_{sup}$	$T_{b}$	521.4 ± 23.0	24.43	5.30	29.79	0.96	0.97 ***
	$T_{s_{barsi}}$	484.7 ± 23.0	30.04	5.53	40.19	0.93	0.94 ***
	$T_{s_{SC}}$	477.6 ± 23.0	35.12	6.44	46.76	0.90	0.93 ***
	$T_{s_{RTE}}$	478.9 ± 22.0	33.65	6.16	44.94	0.91	0.93 ***
	$T_{s_{SW}}$	479.0 ± 22.0	36.965	6.82	49.05	0.89	0.90 ***

**Table 7:** Average (±95% confidence interval) of the measured soil heat flux (G; W m^−2^) in the flux towers, and the average (±95% confidence interval), mean absolute error (*MAE*), mean absolute percent error (*MAPE*), root mean square error (*RMSE*), Willmott coefficient (d), and Pearson correlation coefficient (r) of the estimated soil heat flux using the conventional ($a_{con}$) and parameterized ($a_{sup}$) surface albedo models combined with brightness temperature ($T_{b}$) and surface temperature corrected by the Barsi model ($T_{s_{barsi}}$), the single-channel model ($T_{s_{SC}}$), the radiative transfer equation model ($T_{s_{RTE}}$), and the split-window model ($T_{s_{SW}}$). Values with (**) indicate *p*-value < 0.01.

		Average ± IC	*MAE*	*MAPE*	*RMSE*	d	
		W m^−2^	W m^−2^	%	W m^−2^		
Measured G		47.2 ± 6.2					
**Model Combination**							
$a_{con}$	$T_{b}$	63.8 ± 4.4	18.22	56.76	21.54	0.59	0.57 **
	$T_{s_{barsi}}$	71.3 ± 5.3	24.18	73.35	27.89	0.54	0.58 **
	$T_{s_{SC}}$	72.1 ± 5.2	25.87	75.85	28.86	0.53	0.57 **
	$T_{s_{RTE}}$	71.7 ± 5.0	25.60	75.26	28.63	0.53	0.57 **
	$T_{s_{SW}}$	72.1 ± 5.1	25.46	74.85	28.60	0.53	0.57 **
$a_{sup}$	$T_{b}$	63.6 ± 4.5	18.26	56.65	21.47	0.53	0.55 **
	$T_{s_{barsi}}$	72.0 ± 5.2	24.79	74.64	28.41	0.54	0.57 **
	$T_{s_{SC}}$	72.8 ± 5.1	25.87	77.58	29.54	0.53	0.55 **
	$T_{s_{RTE}}$	73.0 ± 5.5	25.60	76.88	29.25	0.53	0.55 **
	$T_{s_{SW}}$	72.6 ± 5.3	25.46	76.43	29.29	0.53	0.58 **

**Table 8:** Average (±95% confidence interval) of the measured sensible heat flux (H; W m^−2^) in the flux towers, and the average (±95% confidence interval), mean absolute error (*MAE*), mean absolute percent error (*MAPE*), root mean square error (*RMSE*), Willmott coefficient (d), and Pearson correlation coefficient (r) of the estimated sensible heat flux using the conventional ($a_{con}$) and parameterized ($a_{sup}$) surface albedo models combined with brightness temperature ($T_{b}$) and surface temperature corrected by the Barsi model ($T_{s_{barsi}}$), the single-channel model ($T_{s_{SC}}$), the radiative transfer equation model ($T_{s_{RTE}}$), and the split-window model ($T_{s_{SW}}$). Values with (***) indicate *p*-value < 0.001.

		Average ± IC	*MAE*	*MAPE*	*RMSE*	d	r
		W m^−2^	W m^−2^	%	W m^−2^		
Measured H		201.7 ± 23.0					
**Model Combination**							
$a_{con}$	$T_{b}$	167.0 ± 18.3	30.27	15.37	36.50	0.84	0.87 ***
	$T_{s_{barsi}}$	148.3 ± 17.0	45.20	22.46	51.94	0.72	0.86 ***
	$T_{s_{SC}}$	140.0 ± 17.5	53.00	26.10	60.73	0.65	0.81 ***
	$T_{s_{RTE}}$	141.8 ± 17.0	51.00	25.47	58.56	0.66	0.82 ***
	$T_{s_{SW}}$	131.0 ± 23.5	64.13	32.28	74.49	0.61	0.69 ***
$a_{sup}$	$T_{b}$	211.2 ± 23.0	28.17	15.06	31.39	0.91	0.84 ***
	$T_{s_{barsi}}$	188.5 ± 21.0	23.66	12.52	30.58	0.90	0.85 ***
	$T_{s_{SC}}$	183.1 ± 21.0	28.38	14.57	36.76	0.86	0.81 ***
	$T_{s_{RTE}}$	186.3 ± 20.0	27.14	14.02	35.46	0.87	0.81 ***
	$T_{s_{SW}}$	170.9 ± 22.0	36.83	18.8	47.00	0.80	0.78 ***

**Table 9:** Average (±95% confidence interval) of the measured latent heat flux (LE; W m^−2^) in the flux towers, and the average (±95% confidence interval), mean absolute error (*MAE*), mean absolute percent error (*MAPE*), root mean square error (*RMSE*), Willmott coefficient (d), and Pearson correlation coefficient (r) of the estimated latent heat flux using the conventional ($a_{con}$) and parameterized ($a_{sup}$) surface albedo models combined with brightness temperature ($T_{b}$) and surface temperature corrected by the Barsi model ($T_{s_{barsi}}$), the single-channel model ($T_{s_{SC}}$), the radiative transfer equation model ($T_{s_{RTE}}$), and the split-window model ($T_{s_{SW}}$). Values with (***) indicate *p*-value < 0.001.

		Average ± IC	*MAE*	*MAPE*	*RMSE*	d	r
		W m^−2^	W m^−2^	%	W m^−2^		
Measured LE		259.5 ± 46.0					
**Models Combination**							
$a_{con}$	$T_{b}$	266.4 ± 41.0	29.42	11.59	37.93	0.95	0.93 ***
	$T_{s_{barsi}}$	247.1 ± 41.0	37.33	13.70	47.45	0.93	0.91 ***
	$T_{s_{SC}}$	248.7 ± 42.0	40.79	15.08	51.01	0.92	0.89 ***
	$T_{s_{RTE}}$	250.2 ± 42.0	41.39	15.23	51.80	0.92	0.88 ***
	$T_{s_{SW}}$	259.0 ± 44.0	41.28	14.75	52.58	0.92	0.86 ***
$a_{sup}$	$T_{b}$	276.6 ± 45.0	29.87	12.87	35.71	0.96	0.95 ***
	$T_{s_{barsi}}$	260.3 ± 43.0	27.59	11.70	33.83	0.97	0.94 ***
	$T_{s_{SC}}$	257.2 ± 43.0	30.04	12.76	37.97	0.96	0.93 ***
	$T_{s_{RTE}}$	257.7 ± 41.0	30.51	12.87	38.71	0.96	0.92 ***
	$T_{s_{SW}}$	270.6 ± 46.0	37.26	14.85	46.64	0.94	0.90 ***

**Table 10:** Average (±95% confidence interval) of the measured evapotranspiration (ET; mm d^−1^) in the flux towers, and the average (±95% confidence interval), mean absolute error (*MAE*), mean absolute percent error (*MAPE*), root mean square error (*RMSE*), Willmott coefficient (d), and Pearson correlation coefficient (r) of the estimated soil heat flux using the conventional ($a_{con}$) and parameterized ($a_{sup}$) surface albedo models combined with brightness temperature ($T_{b}$) and surface temperature corrected by the Barsi model ($T_{s_{barsi}}$), the single-channel model ($T_{s_{SC}}$), the radiative transfer equation model ($T_{s_{RTE}}$), and the split-window model ($T_{s_{SW}}$). Values with (***) indicate *p*-value < 0.001.

		Average ± IC	*MAE*	*MAPE*	*RMSE*	d	r
		W m^−2^	W m^−2^	%	W m^−2^		
Measured ET		3.00 ± 0.50					
**Model Combination**							
$a_{con}$	$T_{b}$	2.69 ± 0.38	0.42	13.07	0.50	0.92	0.95 ***
	$T_{s_{barsi}}$	2.72 ± 0.40	0.39	12.45	0.46	0.94	0.95 ***
	$T_{s_{SC}}$	2.79 ± 0.42	0.38	12.23	0.43	0.94	0.94 ***
	$T_{s_{RTE}}$	2.76 ± 0.39	0.39	12.48	0.45	0.94	0.94 ***
	$T_{s_{SW}}$	3.12 ± 0.49	0.35	13.23	0.44	0.95	0.92 ***
$a_{sup}$	$T_{b}$	2.90 ± 0.39	0.32	11.36	0.37	0.96	0.95 ***
	$T_{s_{barsi}}$	2.95 ± 0.43	0.29	10.98	0.35	0.96	0.95 ***
	$T_{s_{SC}}$	3.05 ± 0.44	0.28	11.14	0.35	0.96	0.94 ***
	$T_{s_{RTE}}$	3.00 ± 0.42	0.30	12.42	0.35	0.96	0.94 ***
	$T_{s_{SW}}$	3.18 ± 0.47	0.35	13.23	0.44	0.95	0.92 ***

## Supplementary Materials

The following are available online at https://www.mdpi.com/article/10.3390/s21217196/s1, Table S1: Average (±95% confidence interval) of the measured net radiation (Rn; W m^−2^), and the average (±95% confidence interval), mean absolute error (*MAE*), mean absolute percent error (*MAPE*), root mean square error (*RMSE*), Willmott coefficient (d) and Pearson correlation coefficient (r) of the estimated net radiation in BPE and FMI using conventional ($a_{con}$), parameterized ($a_{sup}$) surface albedo model combined with brightness temperature ($T_{b}$) and surface temperature corrected by Barsi model ($T_{s_{barsi}}$), single-channel model ($T_{s_{SC}}$), radiative transfer equation model ($T_{s_{RTE}}$) and Split-window model ($T_{s_{SW}}$). Values with (*) indicate *p*-value < 0.05, (**) *p*-value < 0.01 and (***) *p*-value < 0.001. Table S2. Average (±95% confidence interval) of the measured soil heat flux (G; W m^−2^), and the average (±95% confidence interval), mean absolute error (*MAE*), mean absolute percent error (*MAPE*), root mean square error (*RMSE*), Willmott coefficient (d) and Pearson correlation coefficient (r) of the estimated soil heat flux in FMI using conventional ($a_{con}$), parameterized ($a_{sup}$) surface albedo model combined with brightness temperature ($T_{b}$) and surface temperature corrected by Barsi model ($T_{s_{barsi}}$), single-channel model ($T_{s_{SC}}$), radiative transfer equation model ($T_{s_{RTE}}$) and Split-window model ($T_{s_{SW}}$). Values with (*) indicate *p*-value < 0.05, (**) *p*-value < 0.01 and (***) *p*-value < 0.001. Table S3. Average (±95% confidence interval) of the measured sensible heat flux (H; W m^−2^), and the average (±95% confidence interval), mean absolute error (*MAE*), mean absolute percent error (*MAPE*), root mean square error (*RMSE*), Willmott coefficient (d) and Pearson correlation coefficient (r) of the estimated sensible heat flux in BPE and FMI using conventional ($a_{con}$), parameterized ($a_{sup}$) surface albedo model combined with brightness temperature ($T_{b}$) and surface temperature corrected by Barsi model ($T_{s_{barsi}}$), single-channel model ($T_{s_{SC}}$), radiative transfer equation model ($T_{s_{RTE}}$) and Split-window model ($T_{s_{SW}}$). Values with (*) indicate *p*-value < 0.05, (**) *p*-value < 0.01 and (***) *p*-value < 0.001. Table S4. Average (±95% confidence interval) of the measured latent heat flux (LE; W m^−2^), and the average (±95% confidence interval), mean absolute error (*MAE*), mean absolute percent error (*MAPE*), root mean square error (*RMSE*), Willmott coefficient (d) and Pearson correlation coefficient (r) of the estimated latent heat flux in BPE and FMI using conventional ($a_{con}$), parameterized ($a_{sup}$) surface albedo model combined with brightness temperature ($T_{b}$) and surface temperature corrected by Barsi model ($T_{s_{barsi}}$), single-channel model ($T_{s_{SC}}$), radiative transfer equation model ($T_{s_{RTE}}$) and Split-window model ($T_{s_{SW}}$). Values with (*) indicate *p*-value < 0.05, (**) *p*-value < 0.01 and (***) *p*-value < 0.001. Table S5. Average (±95% confidence interval) of the measured evapotranspiration (ET; mm d^−1^), and the average (±95% confidence interval), mean absolute error (*MAE*), mean absolute percent error (*MAPE*), root mean square error (*RMSE*), Willmott coefficient (d) and Pearson correlation coefficient (r) of the estimated evapotranspiration in BPE and FMI using conventional ($a_{con}$), parameterized ($a_{sup}$) surface albedo model combined with brightness temperature ($T_{b}$) and surface temperature corrected by Barsi model ($T_{s_{barsi}}$), single-channel model ($T_{s_{SC}}$), radiative transfer equation model ($T_{s_{RTE}}$) and Split-window model ($T_{s_{SW}}$). Values with (*) indicate *p*-value < 0.05, (**) *p*-value < 0.01 and (***) *p*-value < 0.001.
