# Supplementary material for: Surface Albedo and Temperature Models for Surface Energy Balance Fluxes and Evapotranspiration Using SEBAL and Landsat 8 over Cerrado-Pantanal, Brazil
Source: Sensors (Basel). 2021 Oct 29;21(21):7196. doi: 10.3390/s21217196 (PMC8587917; doi:10.3390/s21217196)
Supplement: Supplementary file 1 [file sensors-21-07196-s001.zip › sensors-1375038-supplementary.pdf]

Surface Albedo and Temperature Models for Surface Energy Balance Fluxes and Evapotranspiration using SEBAL and Landsat 8 over Cerrado-Pantanal, Brazil

Lucas Peres Angelini [1], Marcelo Sacardi Biudes [2,*], Nadja Gomes Machado [3], Hatim M. E. Geli [4,*], George Louis Vourlitis [4], Anderson Ruhoff [5], José de Souza Nogueira [6]

Supplementary Material

Table S1. Average (±95% confidence interval) of the measured net radiation (Rn; W m^{-2}), and the average (±95% confidence interval), mean absolute error (MAE), mean absolute percent error (MAPE), root mean square error (RMSE), Willmott coefficient (d) and Pearson correlation coefficient (r) of the estimated net radiation in BPE and FMI using conventional (a_{con}), parameterized (a_{sup}) surface albedo model combined with brightness temperature (T_b) and surface temperature corrected by Barsi model ($T_{s_{barsi}}$), single-channel model ($T_{s_{SC}}$), radiative transfer equation model ($T_{s_{RTE}}$) and Split-window model ($T_{s_{SW}}$). Values with (*) indicate p-value < 0.05, (**) p-value < 0.01 and (***) p-value < 0.001.

Site	Models		Avg±IC	MAE	MAPE	RMSE	d	r
BPE	Rn Measured		537.8 ± 33.2					
	a_{con}	T_b	511.9 ± 36.1	26.2	5.0	29.0	0.92	0.97***
		$T_{s_{barsi}}$	492.0 ± 34.1	45.9	8.6	50.2	0.79	0.91**
		$T_{s_{SC}}$	488.5 ± 32.9	49.3	9.2	54.1	0.76	0.89**
		$T_{s_{RTE}}$	489.7 ± 34.8	48.1	9.0	53.2	0.77	0.89**
		$T_{s_{SW}}$	493.1 ± 32.6	44.7	8.3	49.3	0.78	0.90**
	a_{sup}	T_b	539.1 ± 38.0	11.2	2.1	13.3	0.98	0.98***
		$T_{s_{barsi}}$	519.1 ± 37.7	22.3	4.2	25.7	0.93	0.95***
		$T_{s_{SC}}$	515.7 ± 34.7	24.3	4.6	29.1	0.92	0.93***
		$T_{s_{RTE}}$	516.9 ± 36.6	24.3	4.6	28.5	0.92	0.93***
		$T_{s_{SW}}$	520.3 ± 34.2	22.2	4.2	24.9	0.94	0.94***
FMI	Rn Measured		495.9 ± 34.3					
	a_{con}	T_b	464.9 ± 26.7	33.9	6.1	45.3	0.92	0.97***
		$T_{s_{barsi}}$	424.7 ± 25.2	69.9	13.1	82.5	0.77	0.91***
		$T_{s_{SC}}$	417.0 ± 25.9	77.3	14.6	91.0	0.73	0.89***
		$T_{s_{RTE}}$	419.0 ± 25.2	75.4	14.2	88.7	0.74	0.90***
		$T_{s_{SW}}$	418.2 ± 25.1	78.9	14.8	92.7	0.71	0.85***
	a_{sup}	T_b	511.7 ± 26.3	28.1	6.2	32.9	0.96	0.98***
		$T_{s_{barsi}}$	471.5 ± 24.1	32.2	5.9	43.4	0.93	0.96***
		$T_{s_{SC}}$	463.9 ± 22.7	38.1	7.0	50.6	0.90	0.95***
		$T_{s_{RTE}}$	465.8 ± 24.4	36.2	6.6	48.5	0.91	0.95***
		$T_{s_{SW}}$	465.0 ± 22.4	41.0	7.5	53.8	0.88	0.92***

Table S2. Average (±95% confidence interval) of the measured soil heat flux (G; W m^{-2}), and the average (±95% confidence interval), mean absolute error (MAE), mean absolute percent error (MAPE), root mean square error (RMSE), Willmott coefficient (d) and Pearson correlation coefficient (r) of the estimated soil heat flux in FMI using conventional (a_{con}), parameterized (a_{sup}) surface albedo model combined with brightness temperature (T_b) and surface temperature corrected by Barsi model ($T_{s_{barsi}}$), single-channel model ($T_{s_{SC}}$), radiative transfer equation model ($T_{s_{RTE}}$) and Split-window model ($T_{s_{SW}}$). Values with (*) indicate p-value < 0.05, (**) p-value < 0.01 and (***) p-value < 0.001.

Site	Models		Avg±IC	MAE	MAPE	RMSE	d	r
FMI	G Measured		47.3 ± 6.5					
	a_{con}	T_b	63.6 ± 4.4	18.2	56.8	21.5	0.59	0.56**
		$T_{s_{barsi}}$	71.1 ± 4.9	24.2	73.4	27.9	0.54	0.55**
		$T_{s_{SC}}$	72.1 ± 5.2	25.0	75.9	28.9	0.53	0.55**
		$T_{s_{RTE}}$	71.9 ± 5.1	24.8	75.3	28.6	0.54	0.55**
		$T_{s_{SW}}$	71.8 ± 5.1	24.7	74.9	28.6	0.54	0.54**
	a_{sup}	T_b	63.5 ± 4.6	18.3	56.7	21.5	0.59	0.56**
		$T_{s_{barsi}}$	71.7 ± 5.2	24.8	74.6	28.4	0.54	0.56**
		$T_{s_{SC}}$	72.9 ± 5.1	25.9	77.6	29.5	0.53	0.55**
		$T_{s_{RTE}}$	72.6 ± 5.1	25.6	76.9	29.3	0.53	0.56**
		$T_{s_{SW}}$	72.6 ± 5.5	25.5	76.4	29.3	0.53	0.55**

Table S3. Average (±95% confidence interval) of the measured sensible heat flux (H; W m^{-2}), and the average (±95% confidence interval), mean absolute error (MAE), mean absolute percent error (MAPE), root mean square error (RMSE), Willmott coefficient (d) and Pearson correlation coefficient (r) of the estimated sensible heat flux in BPE and FMI using conventional (a_{con}), parameterized (a_{sup}) surface albedo model combined with brightness temperature (T_b) and surface temperature corrected by Barsi model ($T_{s_{barsi}}$), single-channel model ($T_{s_{SC}}$), radiative transfer equation model ($T_{s_{RTE}}$) and Split-window model ($T_{s_{SW}}$). Values with (*) indicate p-value < 0.05, (**) p-value < 0.01 and (***) p-value < 0.001.

Site	Models		Avg±IC	MAE	MAPE	RMSE	d	r
BPE	H Measured		213.5 ± 33.5					
	a_{con}	T_b	175.8 ± 21.2	37.8	16.5	45.5	0.70	0.87*
		$T_{s_{barsi}}$	164.7 ± 21.2	48.8	22.0	53.7	0.66	0.91**
		$T_{s_{SC}}$	149.2 ± 19.8	64.3	29.2	69.5	0.57	0.87*
		$T_{s_{RTE}}$	151.4 ± 19.9	62.1	28.1	67.6	0.57	0.86*
		$T_{s_{SW}}$	141.7 ± 39.1	71.8	34.7	78.6	0.62	0.81*
	a_{sup}	T_b	195.5 ± 23.9	26.0	11.6	30.6	0.85	0.86*
		$T_{s_{barsi}}$	180.1 ± 24.0	33.4	14.7	39.8	0.77	0.91**
		$T_{s_{SC}}$	169.3 ± 21.1	44.2	19.6	50.5	0.68	0.88**
		$T_{s_{RTE}}$	169.3 ± 21.4	44.2	19.6	50.5	0.68	0.88**
		$T_{s_{SW}}$	160.6 ± 44.7	53.0	25.8	61.8	0.72	0.83*
FMI	H Measured		178.0 ± 28.3					
	a_{con}	T_b	159.4 ± 27.0	25.0	14.6	28.6	0.90	0.89***
		$T_{s_{barsi}}$	135.5 ± 24.6	42.7	22.8	50.7	0.72	0.82**
		$T_{s_{SC}}$	132.9 ± 22.5	45.1	23.9	53.7	0.70	0.79**
		$T_{s_{RTE}}$	135.0 ± 23.5	43.2	23.0	51.3	0.72	0.82**
		$T_{s_{SW}}$	119.2 ± 23.5	58.7	30.6	71.5	0.56	0.57*
	a_{sup}	T_b	203.1 ± 34.8	30.8	18.8	33.3	0.90	0.92***
		$T_{s_{barsi}}$	181.1 ± 31.4	16.6	11.7	21.3	0.95	0.91***
		$T_{s_{SC}}$	178.9 ± 29.0	17.2	11.9	21.2	0.95	0.90***
		$T_{s_{RTE}}$	178.9 ± 28.1	17.2	11.9	21.2	0.95	0.90***
		$T_{s_{SW}}$	164.3 ± 26.3	24.0	14.4	33.4	0.86	0.78**

Table S4. Average (±95% confidence interval) of the measured latent heat flux (LE; W m^{-2}), and the average (±95% confidence interval), mean absolute error (MAE), mean absolute percent error (MAPE), root mean square error (RMSE), Willmott coefficient (d) and Pearson correlation coefficient (r) of the estimated latent heat flux in BPE and FMI using conventional (a_{con}), parameterized (a_{sup}) surface albedo model combined with brightness temperature (T_b) and surface temperature corrected by Barsi model ($T_{s_{barsi}}$), single-channel model ($T_{s_{SC}}$), radiative transfer equation model ($T_{s_{RTE}}$) and Split-window model ($T_{s_{SW}}$). Values with (*) indicate p-value < 0.05, (**) p-value < 0.01 and (***) p-value < 0.001.

Site	Models		Avg±IC	MAE	MAPE	RMSE	d	r
BPE	LE Measured		324.3 ± 60.7					
	a_{con}	T_b	329.7 ± 38.0	28.6	10.1	36.2	0.93	0.93**
		$T_{s_{barsi}}$	310.4 ± 42.1	34.4	10.9	40.2	0.91	0.93**
		$T_{s_{SC}}$	319.8 ± 36.7	38.9	13.0	43.4	0.88	0.89**
		$T_{s_{RTE}}$	319.5 ± 36.0	39.7	13.2	44.3	0.88	0.88**
		$T_{s_{SW}}$	337.2 ± 45.1	41.6	13.3	48.4	0.88	0.82*
	a_{sup}	T_b	351.8 ± 44.8	37.9	13.6	42.6	0.91	0.93**
		$T_{s_{barsi}}$	338.0 ± 41.8	29.5	10.8	36.3	0.93	0.94**
		$T_{s_{SC}}$	342.9 ± 39.6	33.9	12.6	43.5	0.90	0.90**
		$T_{s_{RTE}}$	342.5 ± 39.9	34.4	12.7	44.1	0.89	0.89**
		$T_{s_{SW}}$	361.4 ± 53.6	53.3	17.5	59.8	0.85	0.82*
FMI	LE Measured		232.2 ± 61.0					
	a_{con}	T_b	221.5 ± 50.5	23.8	11.7	29.8	0.97	0.94***
		$T_{s_{barsi}}$	203.1 ± 46.1	30.9	14.2	38.6	0.94	0.93***
		$T_{s_{SC}}$	198.9 ± 44.0	32.9	14.9	41.2	0.93	0.92***
		$T_{s_{RTE}}$	198.5 ± 45.4	33.3	15.0	42.3	0.93	0.92***
		$T_{s_{SW}}$	204.0 ± 45.1	29.9	13.6	34.3	0.95	0.94***
	a_{sup}	T_b	247.5 ± 54.0	27.7	14.8	34.0	0.96	0.95***
		$T_{s_{barsi}}$	229.7 ± 50.0	24.6	12.3	30.1	0.97	0.94***
		$T_{s_{SC}}$	225.4 ± 49.6	24.7	12.3	30.5	0.96	0.94***
		$T_{s_{RTE}}$	224.8 ± 50.8	25.3	12.4	31.6	0.96	0.93***
		$T_{s_{SW}}$	231.8 ± 46.8	22.4	12.2	28.8	0.97	0.95***

Table S5. Average (±95% confidence interval) of the measured evapotranspiration (ET; mm d^{-1}), and the average (±95% confidence interval), mean absolute error (MAE), mean absolute percent error (MAPE), root mean square error (RMSE), Willmott coefficient (d) and Pearson correlation coefficient (r) of the estimated evapotranspiration in BPE and FMI using conventional (a_{con}), parameterized (a_{sup}) surface albedo model combined with brightness temperature (T_b) and surface temperature corrected by Barsi model ($T_{s_{barsi}}$), single-channel model ($T_{s_{SC}}$), radiative transfer equation model ($T_{s_{RTE}}$) and Split-window model ($T_{s_{SW}}$). Values with (*) indicate p-value < 0.05, (**) p-value < 0.01 and (***) p-value < 0.001.

Site	Models		Avg±IC	MAE	MAPE	RMSE	d	r
BPE	ET Measured		3.3 ± 0.6					
	a_{con}	T_b	3.0 ± 0.4	0.4	10.7	0.5	0.89	0.97***
		$T_{s_{barsi}}$	3.0 ± 0.4	0.4	10.5	0.4	0.91	0.97***
		$T_{s_{SC}}$	3.1 ± 0.5	0.3	10.3	0.4	0.93	0.94**
		$T_{s_{RTE}}$	3.1 ± 0.5	0.4	10.6	0.4	0.92	0.94**
		$T_{s_{SW}}$	3.4 ± 0.6	0.3	8.7	0.4	0.95	0.91**
	a_{sup}	T_b	3.2 ± 0.3	0.4	12.7	0.5	0.87	0.92**
		$T_{s_{barsi}}$	3.2 ± 0.3	0.4	12.2	0.4	0.90	0.93**
		$T_{s_{SC}}$	3.3 ± 0.3	0.4	14.0	0.5	0.87	0.91*
		$T_{s_{RTE}}$	3.4 ± 0.3	0.5	17.4	0.5	0.83	0.84*
		$T_{s_{SW}}$	3.4 ± 0.3	0.3	11.0	0.4	0.92	0.90*
FMI	ET Measured		2.8 ± 0.7					
	a_{con}	T_b	2.5 ± 0.5	0.4	14.7	0.5	0.93	0.95***
		$T_{s_{barsi}}$	2.6 ± 0.6	0.4	13.8	0.5	0.95	0.95***
		$T_{s_{SC}}$	2.6 ± 0.6	0.4	13.6	0.5	0.95	0.95***
		$T_{s_{RTE}}$	2.5 ± 0.6	0.4	13.8	0.5	0.94	0.95***
		$T_{s_{SW}}$	3.0 ± 0.7	0.4	16.4	0.5	0.95	0.92***
	a_{sup}	T_b	2.6 ± 0.4	0.2	7.5	0.3	0.98	0.97***
		$T_{s_{barsi}}$	2.7 ± 0.4	0.2	8.1	0.3	0.98	0.97***
		$T_{s_{SC}}$	2.7 ± 0.4	0.2	7.9	0.3	0.98	0.97***
		$T_{s_{RTE}}$	2.6 ± 0.4	0.2	8.1	0.3	0.98	0.97***
		$T_{s_{SW}}$	2.7 ± 0.3	0.3	12.9	0.4	0.94	0.92***
